# Transcriptomic Profiling Reveals Isoform-Specific Regulatory Roles of miR-196A and miR-196B in Colorectal Cancer Cells

**DOI:** 10.3390/ijms27093959

**Published:** 2026-04-29

**Authors:** Ji Su Mo, Dong Seok Shin, Youn Ho Han

**Affiliations:** Department of Oral Pharmacology, College of Dentistry, Wonkwang University, Iksan 54538, Republic of Korea; siuale97@hanmail.net (J.S.M.);

**Keywords:** miR-196A, miR-196B, colorectal cancer, isoform-specific, transcriptomics

## Abstract

MicroRNAs (miRNAs) play important roles in the regulation of gene expression and are frequently dysregulated in cancer. Among them, the miR-196 family has been implicated in multiple malignancies, including colorectal cancer (CRC), but the isoform-specific transcriptional effects of miR-196A and miR-196B remain poorly understood. In this study, we generated miR-196A and miR-196B knockout SW48 CRC cell lines using CRISPR-based genome editing and performed RNA sequencing to investigate the transcriptional consequences of individual miR-196 isoform deletion. Transcriptomic analysis revealed widespread gene expression changes in both knockout models and demonstrated distinct clustering patterns between parental SW48 cells and miR-196-deficient cells. Functional enrichment analysis indicated that the altered genes were associated with biological processes related to cytoskeletal organization, intracellular transport, protein folding, and metabolic regulation. Notably, both shared and isoform-specific transcriptional alterations were observed, suggesting that miR-196A and miR-196B contribute to partially overlapping but distinct regulatory networks in CRC cells. Collectively, these findings provide a comprehensive transcriptomic overview of miR-196 isoform deletion in colorectal cancer cells and highlight potential isoform-dependent transcriptional programs that may contribute to CRC biology.

## 1. Introduction

Colorectal cancer (CRC) is the third most common malignancy and the second leading cause of cancer-related death worldwide, with more than one million new cases diagnosed annually [1]. Despite significant advances in surgical techniques, chemotherapy, and targeted therapies, the overall prognosis for patients with advanced or metastatic CRC remains poor, underscoring the need for a deeper understanding of the molecular mechanisms driving disease initiation and progression [2]. CRC development involves the accumulation of complex genetic and epigenetic alterations that dysregulate key signaling pathways controlling cell proliferation, apoptosis, differentiation, and invasion [3].

MicroRNAs (miRNAs) are a class of small, non-coding RNAs approximately 20–24 nucleotides in length that regulate gene expression post-transcriptionally by binding to complementary sequences, typically in the 3′ untranslated regions (3′UTRs) of target mRNAs, leading to translational repression or mRNA degradation [4]. Accumulating evidence has demonstrated that miRNAs function as critical regulators of tumor biology, acting as either oncogenes or tumor suppressors depending on the cellular context and the repertoire of target genes they regulate [5]. Aberrant miRNA expression profiles have been documented across virtually all cancer types, including CRC, where they influence nearly every hallmark of malignancy.

The miR-196 family, which includes miR-196A and miR-196B, represents a group of miRNAs encoded within the HOX gene clusters, specifically at paralogous loci in the *HOXA*, *HOXB*, and *HOXC* clusters [6]. These miRNAs have been reported to regulate HOX gene expression during embryonic development and are known to target key developmental regulators including *HOXB8*, *HOXC8*, *HOXD8*, and *HOXA5* [7,8]. Beyond their developmental roles, dysregulation of miR-196 family members has been implicated in multiple human cancers, including leukemia, gastric cancer, breast cancer, and colorectal cancer [9,10]. In CRC specifically, both miR-196A and miR-196B expression levels are significantly elevated in tumor tissues compared with adjacent normal mucosa, and high co-expression of these two isoforms has been associated with aggressive clinicopathological features, increased metastatic potential, and reduced disease-free and overall survival [11]. Functional studies have identified diverse oncogenic roles for these miRNAs, including promotion of cell migration and invasion via targeting of IκBα and the NF-κB pathway [12], regulation of FAS-mediated apoptosis [13], modulation of cancer stem cell properties through STAT3 signaling [14], and association with liver metastasis [15].

Although miR-196A and miR-196B share highly similar mature sequences and partially overlapping predicted target genes, increasing evidence suggests that individual isoforms may exert distinct biological effects depending on their genomic context, expression levels, and the specific cellular environment in which they operate [16]. The two isoforms are encoded at distinct genomic loci—miR-196A within the *HOXA* and *HOXC* clusters, and miR-196B within the *HOXB* cluster—which may expose them to different transcriptional regulatory mechanisms and lead to differential expression patterns across tissues and disease states. While the oncogenic properties of miR-196A have been characterized in CRC through studies examining specific target genes such as *ING5* and *ZG16* [17,18], isoform-specific transcriptome-wide regulatory programs of miR-196A versus miR-196B in CRC remain poorly understood, particularly at the level of genome-wide transcriptional consequences.

CRISPR/Cas9-based genome editing has emerged as a powerful tool for generating loss-of-function models of miRNA genes, enabling precise deletion of specific miRNA loci and subsequent analysis of the resulting transcriptomic consequences [19]. Unlike transient transfection approaches using antagomirs or sponge constructs, stable CRISPR-based knockout provides a clean genetic background for studying the full spectrum of gene expression changes attributable to a given miRNA, minimizing off-target and context-dependent variability [20]. RNA sequencing combined with systematic functional enrichment analyses further allows for unbiased, genome-wide characterization of the transcriptional programs regulated by individual miRNA isoforms.

In the present study, we generated miR-196A-knockout (SW48-KO196A) and miR-196B-knockout (SW48-KO196B) colorectal cancer cell lines using CRISPR-based genome editing and performed comprehensive transcriptomic analyses by RNA sequencing. By integrating differential gene expression analysis, functional enrichment analysis, hierarchical clustering, and top-gene profiling, we aimed to identify both shared and isoform-specific transcriptional programs regulated by miR-196. Our results provide new insights into the regulatory networks controlled by miR-196 isoforms and reveal both convergent and divergent transcriptional consequences of losing each isoform in a CRC cellular context, with potential implications for understanding isoform-specific contributions to CRC biology.

## 2. Results

### 2.1. Generation and Validation of miR-196A and miR-196B Knockout SW48 Cells

**Figure 1:**
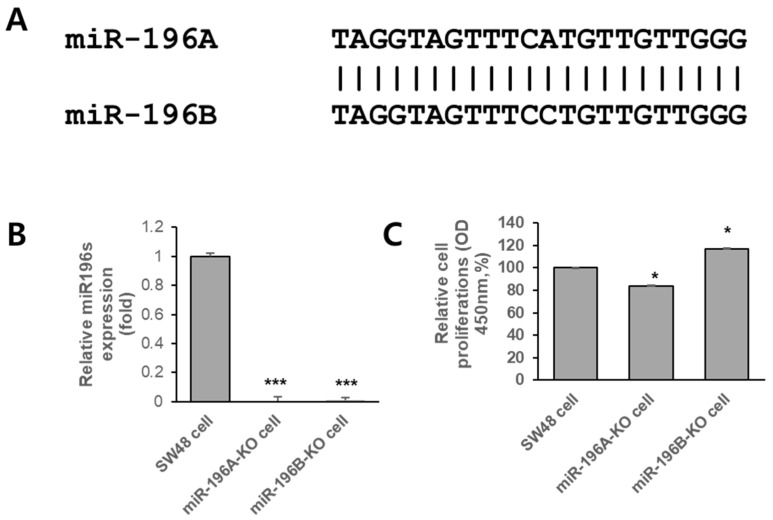
Generation and functional validation of miR-196 knockout SW48 cells. (**A**) Sequence alignment of miR-196A and miR-196B showing a high degree of similarity between the two miRNA isoforms. (**B**) Quantitative RT-PCR analysis of miR-196 expression in parental SW48 cells and in miR-196A-KO or miR-196B-KO cells. Relative expression levels were normalized to an internal control RNA and presented as fold change compared with control cells. (**C**) Cell proliferation assay showing the relative growth rates of control, miR-196A-KO, and miR-196B-KO SW48 cells. Data represent the mean ± SD from independent experiments. Statistical significance is indicated (* *p* < 0.05, *** *p* < 0.001).

To investigate the functional roles of miR-196 isoforms in colorectal cancer, miR-196A- and miR-196B-knockout SW48 cell lines were generated. The sequence comparison between miR-196A and miR-196B revealed a high degree of similarity, differing only at a single nucleotide position (Figure 1A), suggesting that the two isoforms may share overlapping biological functions. To validate the successful deletion of miR-196 isoforms, the expression levels of miR-196A and miR-196B were measured by quantitative RT-PCR. As shown in Figure 1B, the expression levels of miR-196 were significantly reduced in both miR-196A-KO and miR-196B-KO cells compared with parental SW48 cells, confirming the effective disruption of miR-196 expression. To examine whether the deletion of miR-196 affects cellular proliferation, cell growth assays were performed. As shown in Figure 1C, miR-196A-KO cells exhibited reduced proliferation compared with control SW48 cells, showing approximately an 18% decrease in proliferative activity, whereas miR-196B-KO cells showed approximately a 15% increase in proliferation relative to the control. These results suggest that the two miR-196 isoforms may exert distinct effects on the regulation of colorectal cancer cell growth.

### 2.2. Identification of Differentially Expressed Genes Following miR-196 Deletion

**Figure 2:**
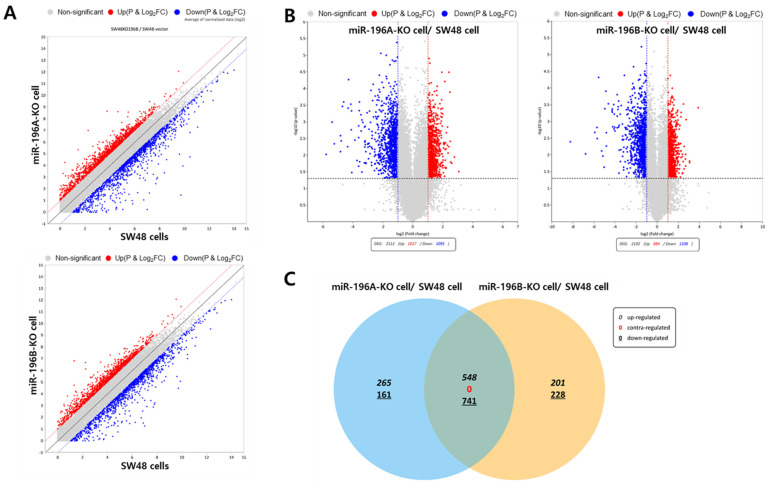
Identification of differentially expressed genes following deletion of miR-196 isoforms in SW48 cells. (**A**) Volcano plot showing the distribution of differentially expressed genes (DEGs) in miR-196 knockout cells compared with control SW48 cells. Significantly up-regulated and down-regulated genes are highlighted based on predefined statistical thresholds. (**B**) Summary of DEGs identified in the transcriptomic analysis, showing the numbers of significantly up-regulated and down-regulated genes in miR-196 knockout cells relative to control cells. The vertical dashed lines indicate the fold-change thresholds, and the horizontal dashed line represents the statistical significance threshold (adjusted *p*-value cutoff). The grey shaded area indicates genes that are not significantly differentially expressed. (**C**) Venn diagram illustrating the overlap of DEGs between miR-196A-KO and miR-196B-KO cells. A subset of genes was commonly regulated by both miR-196 isoforms, while additional genes were uniquely altered in each knockout condition.

To investigate the global transcriptional effects of miR-196 isoforms in colorectal cancer cells, RNA sequencing analysis was performed using miR-196A-KO, miR-196B-KO, and parental SW48 cells. Differential gene expression analysis revealed substantial transcriptomic alterations following deletion of the miR-196 isoforms. As shown in Figure 2A, volcano plot analysis illustrates the overall distribution of significantly up-regulated and down-regulated genes in miR-196 knockout cells relative to control cells. Numerous genes exhibited statistically significant expression changes, indicating that miR-196 plays an important role in regulating gene expression programs in colorectal cancer cells. Quantitative analysis further demonstrated a large number of differentially expressed transcripts in both knockout cell lines (Figure 2B), including genes that were significantly up-regulated or down-regulated compared with parental SW48 cells. These results suggest that the loss of miR-196 induces extensive transcriptional reprogramming. To determine whether miR-196A and miR-196B regulate common or distinct gene sets, overlap analysis was performed using a Venn diagram. As shown in Figure 2C, a subset of genes was commonly altered in both miR-196A-KO and miR-196B-KO cells, suggesting shared regulatory functions between the two isoforms. In contrast, several genes were uniquely regulated in each knockout condition, indicating that miR-196A and miR-196B may also exert isoform-specific transcriptional effects.

### 2.3. Functional Enrichment Analysis of Differentially Expressed Genes in miR-196A-KO and miR-196B-KO SW48 Cells

**Figure 3:**
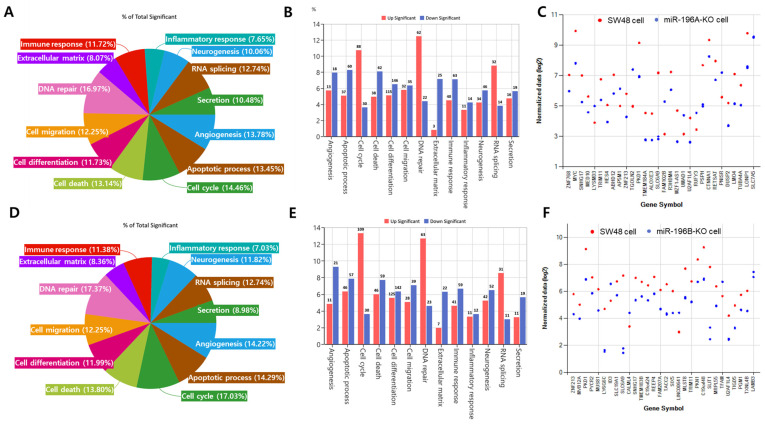
Functional enrichment analysis of differentially expressed genes following miR-196A or miR-196B deletion. (**A**) Functional categorization of differentially expressed genes (DEGs) identified in miR-196A-KO cells compared with control SW48 cells. The pie chart shows the percentage distribution of genes associated with major biological processes. The percentages may exceed 100% because individual genes can be associated with multiple functional categories and are therefore counted in more than one category. (**B**) Distribution of significantly up-regulated and down-regulated genes in each functional category in miR-196A-KO cells. (**C**) Expression profiles of representative genes altered in miR-196A-KO cells. Each point represents normalized RNA-seq expression values from independent samples. (**D**) Functional classification of DEGs identified in miR-196B-KO cells relative to control cells. The percentages may exceed 100% because individual genes can be associated with multiple functional categories and are therefore counted in more than one category. (**E**) Numbers of significantly up-regulated and down-regulated genes in each functional category in miR-196B-KO cells. (**F**) Expression patterns of representative genes affected by miR-196B deletion.

To further explore the biological significance of the transcriptional changes induced by miR-196 deletion, functional enrichment analysis was performed using the differentially expressed genes identified in miR-196A-KO and miR-196B-KO SW48 cells. In miR-196A-KO cells, the DEGs were distributed across several biological processes associated with cancer-related pathways (Figure 3A). These functional categories included cell cycle regulation, apoptotic processes, angiogenesis, DNA repair, immune response, extracellular matrix organization, cell migration, and cell differentiation. Among these categories, genes involved in cell cycle regulation and apoptotic processes represented a substantial proportion of the altered transcripts. This functional classification provides a broad overview of the biological processes potentially affected by miR-196A deletion in colorectal cancer cells. Further analysis revealed both up-regulated and down-regulated genes within each functional category (Figure 3B). Genes associated with DNA repair, apoptosis, and cell cycle regulation showed prominent transcriptional changes following miR-196A deletion. Representative genes displaying significant expression changes are shown in Figure 3C. A similar functional analysis was performed for genes differentially expressed in miR-196B-KO cells. As shown in Figure 3D, the affected genes were also enriched in biological processes associated with cell cycle regulation, apoptosis, DNA repair, immune response, and extracellular matrix organization. The distribution of genes across these categories was broadly comparable to that observed in miR-196A-KO cells, suggesting overlapping biological functions regulated by the two miR-196 isoforms. The numbers of up-regulated and down-regulated genes within each functional category are presented in Figure 3E, and expression patterns of representative genes are shown in Figure 3F. Taken together, these results provide an initial functional landscape of transcriptional alterations associated with the loss of miR-196 isoforms.

### 2.4. Transcriptomic Clustering and Reproducibility of RNA-Seq Data

**Figure 4:**
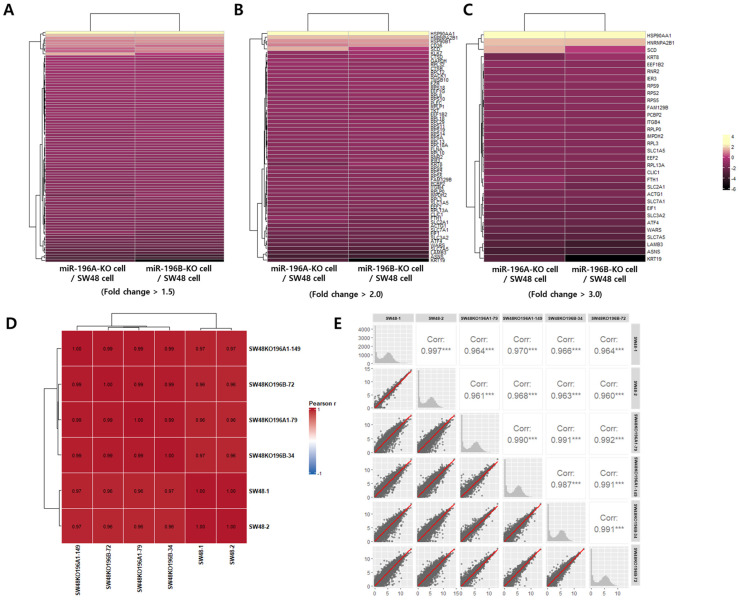
Hierarchical clustering and reproducibility assessment of RNA-seq data from miR-196 knockout SW48 cells. (**A**–**C**) Heatmap visualization of differentially expressed genes between miR-196 knockout cells and parental SW48 cells using fold-change thresholds of >1.5 (**A**), >2.0 (**B**), and >3.0 (**C**). Hierarchical clustering reveals distinct expression patterns associated with miR-196 deletion. (**D**) Pearson correlation heatmap showing strong correlations among biological replicates and experimental groups. Color intensity represents the Pearson correlation coefficient (r), ranging from −1 to 1, where values closer to 1 indicate stronger similarity between samples. The numerical values in each cell correspond to the correlation coefficients. (**E**) Pairwise scatter plot matrix demonstrating high concordance in gene expression profiles across samples. The red line represents the linear regression fit. Asterisks (***) indicate statistical significance (*p* < 0.001).

To further evaluate the transcriptional patterns associated with miR-196 deletion, hierarchical clustering and correlation analyses were performed using the RNA-seq dataset. Heatmap analysis revealed clear expression patterns between miR-196 knockout cells and parental SW48 cells across different fold-change thresholds (Figure 4A–C). When genes with fold changes greater than 1.5, 2.0, and 3.0 were analyzed, distinct clustering patterns were observed, indicating consistent transcriptional alterations following miR-196 deletion. Notably, a large number of genes exhibited coordinated expression changes in both miR-196A-KO and miR-196B-KO cells compared with parental SW48 cells, suggesting that the two miR-196 isoforms share overlapping regulatory effects on gene expression. These clustering patterns support the functional similarities observed in the enrichment analysis and indicate that deletion of the two miR-196 isoforms induces broadly comparable transcriptomic responses. Increasing the fold-change threshold further highlighted a subset of strongly regulated genes that were consistently altered in both knockout conditions. To assess the reproducibility and consistency of the RNA-seq data, Pearson correlation analysis was performed among biological replicates and experimental groups. As shown in Figure 4D, strong correlations were observed across all samples, with Pearson correlation coefficients generally exceeding 0.96, indicating high reproducibility and reliability of the transcriptomic dataset. In addition, pairwise scatter plot analysis confirmed strong linear relationships between samples (Figure 4E). These results confirm that the RNA-seq dataset is highly reproducible and suitable for subsequent functional and pathway analyses.

### 2.5. Functional Enrichment Analysis of Differentially Expressed Genes

**Figure 5:**
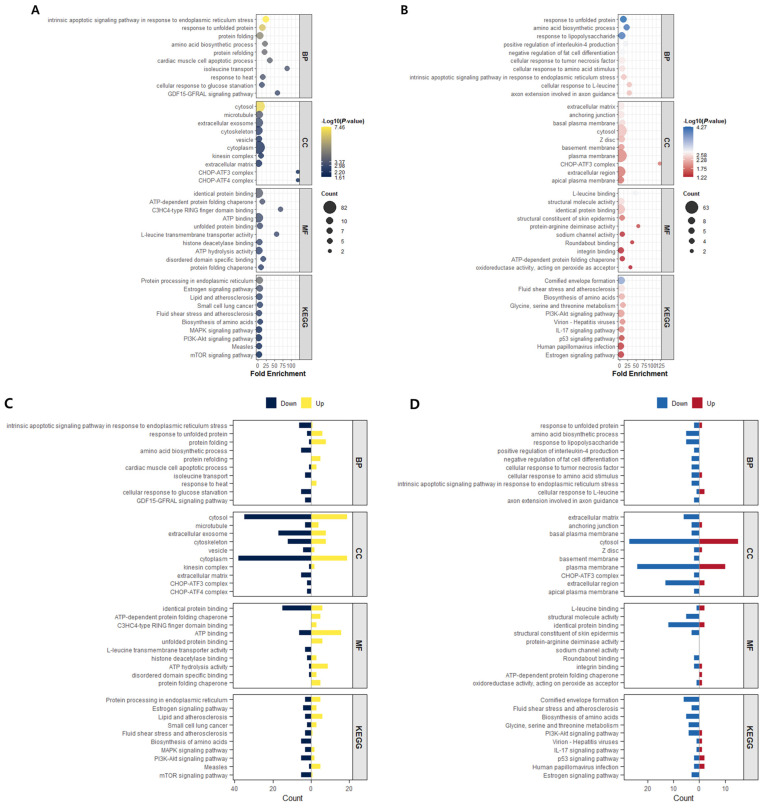
Functional enrichment analysis of genes altered following miR-196 deletion. Gene Ontology (GO) and KEGG pathway enrichment analyses were performed using differentially expressed genes identified from RNA-seq analysis of miR-196A-KO and miR-196B-KO SW48 cells. (**A**) Bubble plot showing significantly enriched GO terms and KEGG pathways in miR-196A-KO cells. Functional categories are grouped into biological process (BP), cellular component (CC), molecular function (MF), and KEGG pathways. Bubble size represents the number of genes associated with each term, and color intensity indicates the −log10(*p*-value). (**B**) Bubble plot showing enriched GO terms and KEGG pathways in miR-196B-KO cells using the same criteria. (**C**) Distribution of up-regulated and down-regulated genes contributing to each enriched functional category in miR-196A-KO cells. (**D**) Distribution of up-regulated and down-regulated genes contributing to each enriched functional category in miR-196B-KO cells.

To further investigate the biological functions associated with transcriptional alterations induced by miR-196 deletion, Gene Ontology (GO) and KEGG pathway enrichment analyses were performed using differentially expressed genes (DEGs) identified from RNA-seq data of miR-196A-KO and miR-196B-KO SW48 cells (Figure 5). While the functional categorization provided a general overview of affected biological processes, GO and KEGG analyses allow a more focused characterization of the molecular pathways associated with miR-196 deletion. In the biological process (BP) category, both miR-196A-KO and miR-196B-KO cells showed significant enrichment of pathways related to response to unfolded protein, protein folding, amino acid biosynthetic processes, and intrinsic apoptotic signaling in response to endoplasmic reticulum stress (Figure 5A,B), suggesting that deletion of either miR-196 isoform affects gene networks involved in endoplasmic reticulum (ER)stress responses and proteostasis regulation. Detailed quantitative enrichment statistics, including gene counts, gene ratios, fold enrichment values, and adjusted *p*-values (FDR), are provided in Supplementary Table S1 for miR-196A-KO and Supplementary Table S2 for miR-196B-KO cells. In the cellular component (CC) category, both knockout cells exhibited enrichment of intracellular structures, including cytosol, microtubule, cytoskeleton, extracellular exosome, vesicle, and cytoplasmic complexes (Figure 5A,B). These results indicate that miR-196 deletion may influence intracellular structural organization and vesicle-mediated transport. Consistent enrichment patterns were also observed in the molecular function (MF) category, where both isoforms showed enrichment of unfolded protein binding, ATP-dependent chaperone activity, histone deacetylase binding, and ATP binding (Figure 5A,B). KEGG pathway analysis further revealed overlapping enrichment of signaling pathways related to protein processing in the endoplasmic reticulum, MAPK signaling, PI3K-Akt signaling, and mTOR signaling (Figure 5A,B), suggesting coordinated regulation of cellular stress and growth signaling pathways. Despite these shared functional patterns, several differences between the two isoforms were also observed. miR-196A-KO cells displayed stronger enrichment of pathways associated with ER stress-mediated apoptosis and metabolic processes, whereas miR-196B-KO cells showed enrichment in processes related to tumor necrosis factor responses and extracellular matrix components (Figure 5A,B). These isoform-specific enrichments suggest that miR-196A and miR-196B contribute to overlapping but partially distinct regulatory networks. The distribution of up-regulated and down-regulated genes within each enriched functional category further illustrates the regulatory patterns associated with each isoform (Figure 5C,D). Collectively, these findings refine the functional overview described above and highlight specific molecular pathways potentially regulated by miR-196 isoforms.

### 2.6. Top Differentially Expressed Genes in miR-196A-KO and miR-196B-KO SW48 Cells

**Figure 6:**
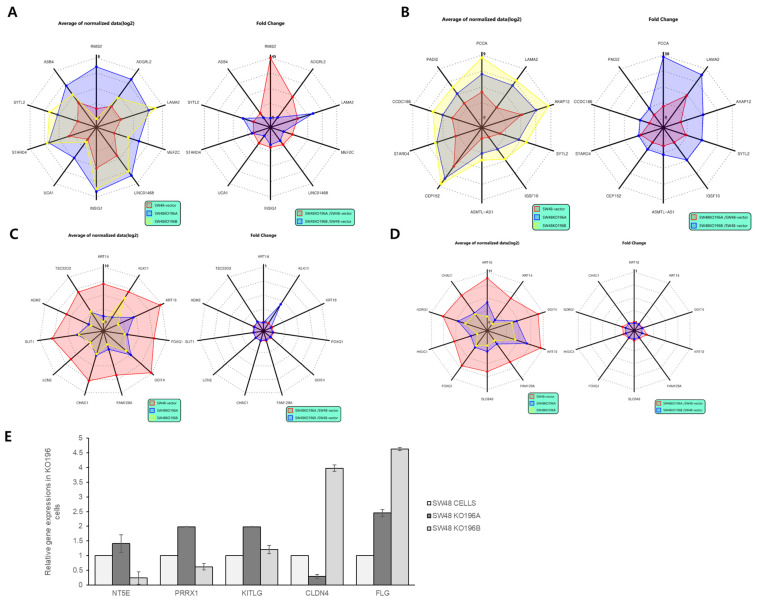
Differential gene expression patterns in miR-196A-KO and miR-196B-KO SW48 cells. Radar charts display the top ten most highly upregulated and downregulated genes in each knockout condition. Left panels show average normalized expression values (log2); right panels show fold change relative to parental SW48 cells (SW48-vector). (**A**) Top ten upregulated genes in miR-196A-KO cells, including *RIMS2*, *ADGRL2*, and *LAMA2*. (**B**) Top ten upregulated genes in miR-196B-KO cells, including *PCCA*, *LAMA2*, and *AKAP12*. (**C**) Top ten downregulated genes in miR-196A-KO cells, including *KRT14*, *KLK11*, and *KRT16*. (**D**) Top ten downregulated genes in miR-196B-KO cells, including *KRT16*, *KRT14*, and *KRT19*. (**E**) qPCR validation of selected miR-196-associated genes (*NT5E*, *PRRX1*, *KITLG*, *CLDN4*, and *FLG*) in parental SW48 and miR-196 isoform knockout cells, showing isoform-dependent differences in gene expression. Data are presented as mean ± SD (*n* = 3).

To further define the transcriptional signatures associated with miR-196 isoform deletion, the top ten most highly upregulated and downregulated genes were identified in each knockout condition and visualized using radar charts displaying both average normalized expression values (log_2_) and fold changes relative to parental SW48 cells (Figure 6). This gene-level analysis complements the functional enrichment results described above (Figure 5) by highlighting specific transcripts that may contribute to the enriched biological pathways. The complete list of top differentially expressed genes and their statistical values is provided in Supplementary Table S3. In miR-196A-KO cells, the most highly upregulated genes included *RIMS2*, *ADGRL2*, *LAMA2*, *MEF2C*, and *INSIG1*, together with several additional transcripts (Figure 6A). Several of these genes are associated with extracellular matrix organization, lipid metabolism, and transcriptional regulation, which is consistent with the enrichment of pathways related to cellular structural organization and metabolic processes observed in the GO and KEGG analyses (Figure 5A). Fold-change analysis further indicated that RIMS2 and ADGRL2 exhibited the most pronounced expression increases compared with control cells. In miR-196B-KO cells, the top upregulated genes included *PCCA*, *LAMA2*, *AKAP12*, *SYTL2*, and *IGSF10*, along with other transcripts showing moderate induction (Figure 6B). Notably, *LAMA2* and *SYTL2* were among the most strongly induced genes in both knockout conditions, suggesting that these genes may represent shared transcriptional targets normally suppressed by miR-196 isoforms. The fold-change radar plots further highlighted *PCCA* and *LAMA2* as the most substantially induced transcripts in miR-196B-KO cells. Many of these genes are involved in cellular metabolism, cytoskeletal organization, and signaling scaffolding, supporting the enrichment of pathways related to intracellular structural components and signaling regulation observed in Figure 5B. Analysis of the most strongly downregulated genes also revealed distinct expression signatures between the two isoforms. In miR-196A-KO cells, prominent downregulated genes included *KRT14*, *KLK11*, *KRT16*, *DDIT4*, and FAM129A (Figure 6C), many of which are associated with epithelial differentiation, stress responses, and inflammatory signaling pathways. Fold-change plots indicated that *KRT14* and *KLK11* exhibited the most substantial decreases relative to control cells. Similarly, miR-196B-KO cells displayed strong downregulation of several epithelial and stress-response genes, including *KRT16*, *KRT14*, *KRT19*, *DDIT4*, and *FAM129A* (Figure 6D). Among these, members of the keratin gene family were particularly prominent, indicating a marked suppression of epithelial structural genes following miR-196B deletion. Taken together, this top-gene analysis reveals both shared and isoform-specific transcriptional changes induced by miR-196 deletion. Several genes, such as *LAMA2*, *SYTL2*, *KRT14*, and *FAM129A*, were commonly affected in both knockout models, supporting the notion that miR-196A and miR-196B regulate overlapping gene networks. At the same time, distinct gene expression patterns observed in each knockout condition suggest isoform-specific regulatory effects, which may contribute to the differential biological pathways identified in the functional enrichment analysis (Figure 5). To provide additional experimental validation of the RNA-seq findings, qPCR analysis was performed for several genes previously reported to be associated with miR-196 regulation, including *NT5E*, *PRRX1*, *KITLG*, *CLDN4*, and *FLG* [16]. The results revealed isoform-dependent differences in gene expression between miR-196A-KO and miR-196B-KO cells (Figure 6E), suggesting that the two miR-196 isoforms may contribute to distinct transcriptional programs.

## 3. Discussion

In this study, CRISPR-based genome editing was used to generate isoform-specific miR-196A and miR-196B knockout SW48 colorectal cancer (CRC) cell lines, followed by RNA sequencing to characterize the transcriptional changes associated with each isoform. Rather than demonstrating direct regulatory effects, the present transcriptomic analysis primarily identifies gene expression alterations associated with the deletion of miR-196 isoforms, revealing both shared and isoform-specific transcriptional patterns. Transcriptomic profiling showed extensive gene expression changes after deletion of either isoform, and the strong Pearson correlation coefficients (>0.96) among biological replicates confirmed the robustness of the RNA-seq dataset. Hierarchical clustering clearly separated KO196A, KO196B, and parental SW48 cells, indicating that disruption of each isoform produces a distinct transcriptional state. These observations are consistent with previous reports showing that CRISPR-mediated miRNA knockout can induce stable transcriptomic remodeling [21,22]. The distinct clustering patterns further suggest that miR-196A and miR-196B may contribute to partially overlapping but non-identical transcriptional programs in CRC cells, despite their sequence similarity and shared genomic origin within HOX clusters.

Functional enrichment analysis indicated that genes altered after miR-196 deletion were enriched in biological processes related to cytoskeletal organization, intracellular transport, protein folding, and metabolic regulation. These pathways are frequently associated with several hallmarks of cancer, including cellular stress adaptation, structural remodeling, and metabolic plasticity. In colorectal cancer, alterations in cytoskeletal organization and intracellular trafficking have been reported to influence cell migration, invasion, and metastatic behavior [23], whereas dysregulation of proteostasis and protein folding pathways can support tumor cell survival under metabolic or oxidative stress conditions [24]. Importantly, these enrichment results should be interpreted as associations with altered transcriptional programs rather than direct functional regulation by miR-196, because the present study focuses primarily on global RNA-seq profiling. Nevertheless, the enrichment of molecular functions such as unfolded protein binding, histone deacetylase binding, and ATP-dependent chaperone activity suggests that miR-196 deletion may be linked to transcriptional changes affecting protein quality control and epigenetic signaling networks in CRC cells.

Among the genes consistently upregulated in both knockout models, *LAMA2* showed one of the most prominent increases. *LAMA2* encodes the α2 chain of laminin, a key component of basement membrane extracellular matrix structures involved in cell adhesion and tissue architecture [25]. Dysregulation of extracellular matrix components plays an important role in colorectal tumor progression and tumor–microenvironment interactions [26]. The increased expression of *LAMA2* following miR-196 deletion therefore suggests a possible association between miR-196 activity and extracellular matrix-related transcriptional programs. Similarly, *SYTL2*, which was also upregulated in both knockout models, is involved in vesicle trafficking and exocytosis [27]. Vesicle transport pathways have recently been recognized as important mediators of tumor cell communication and extracellular vesicle signaling within the tumor microenvironment [28]. The shared upregulation of *SYTL2* in both knockout models may indicate that miR-196 deletion is associated with transcriptional changes affecting membrane trafficking processes. In addition to these shared alterations, isoform-specific changes were observed. In miR-196A-KO cells, *RIMS2* and *ADGRL2* were strongly induced; although these genes are typically associated with neuronal signaling pathways [29,30], neurogenic transcriptional programs have been reported to emerge in epithelial cancers and may contribute to tumor cell plasticity and heterogeneity [31]. The induction of these genes may therefore reflect broader transcriptional plasticity rather than a direct functional shift toward neuronal signaling pathways. In contrast, miR-196B-KO cells showed increased expression of genes including *PCCA* and *AKAP12*. *PCCA* participates in mitochondrial metabolism and amino acid catabolism [32], processes frequently altered during metabolic reprogramming in cancer [33]. *AKAP12* functions as a signaling scaffold protein and has been reported to act as a tumor suppressor in several malignancies [34]. The increased expression of these genes in miR-196B-KO cells suggests that miR-196B deletion may be associated with metabolic and signaling pathway alterations in CRC cells.

A particularly striking observation was the consistent downregulation of multiple keratin genes—including *KRT14*, *KRT16*, and *KRT19*—in both knockout conditions. Keratins are essential structural components of epithelial cells and play key roles in maintaining epithelial integrity and mechanical stability [35]. In colorectal cancer, keratin expression patterns are closely linked to epithelial differentiation status and tumor subtype identity. *KRT14* has been associated with basal-like epithelial phenotypes, while *KRT19* is widely used as a diagnostic marker for epithelial-derived tumors, including CRC [36,37]. The reduction in these keratin transcripts following miR-196 deletion may therefore indicate broader changes in epithelial gene expression programs. Additional downregulated genes included *DDIT4*, *FAM129A*, and *FOXQ1*. *DDIT4* regulates cellular responses to hypoxia and metabolic stress through modulation of mTOR signaling [38], while *FOXQ1* is a transcription factor known to promote epithelial–mesenchymal transition (EMT) and tumor invasion in CRC [39]. The decreased expression of *FOXQ1* following miR-196 deletion suggests that miR-196 activity may be linked to transcriptional programs associated with EMT and tumor cell plasticity, although the current transcriptomic data alone cannot establish direct regulatory relationships.

Several limitations should be considered when interpreting these findings. First, the transcriptomic analysis was performed using a single colorectal cancer cell line (SW48), which may limit the generalizability of the results across diverse CRC models. Future studies incorporating additional CRC cell lines or patient-derived models will be necessary to determine whether the transcriptional patterns observed here are broadly conserved. Despite this limitation, the present study provides a systematic overview of transcriptional alterations associated with miR-196 isoform deletion and highlights both shared and isoform-specific gene expression patterns. These results establish a framework for future functional studies aimed at clarifying how individual miR-196 isoforms contribute to the molecular networks underlying colorectal cancer progression.

## 4. Materials and Methods

### 4.1. Cell Culture

The human colorectal cancer cell line SW48 (ATCC Cat. No. CCL-231) was obtained from the American Type Culture Collection (ATCC, Manassas, VA, USA). Cells were maintained in Dulbecco’s Modified Eagle Medium (DMEM; Gibco, Thermo Fisher Scientific, Waltham, MA, USA) supplemented with 10% fetal bovine serum (FBS; Gibco) and 1% penicillin–streptomycin (Gibco) at 37 °C in a humidified atmosphere containing 5% CO_2_. Cells were routinely passaged at 70–80% confluency and periodically tested to confirm the absence of mycoplasma contamination.

### 4.2. CRISPR/Cas9-Mediated Knockout of miR-196A and miR-196B

To generate miR-196A- and miR-196B-specific knockout cell lines, guide RNAs (gRNAs) targeting the genomic loci of each miRNA precursor were designed using the CRISPOR web tool. The gRNA sequences used were as follows: miR-196A, 5′-TAGGTAGTTTCATGTTGTTGGG-3′; miR-196B, 5′-TAGGTAGTTTCCTGTTGTTGGG-3′. The gRNA oligonucleotides were cloned into the pX459 vector (pSpCas9(BB)-2A-Puro; Addgene plasmid #62988, Watertown, MA, USA), which expresses Cas9 nuclease and a puromycin resistance cassette. SW48 cells were transfected with the constructed plasmids using Lipofectamine 3000 (Thermo Fisher Scientific) according to the manufacturer’s instructions. At 48 h post-transfection, cells were subjected to puromycin selection (1.5 μg/mL) for 3 days to enrich for transfected cells. Single-cell clones were subsequently isolated by limiting dilution and expanded for genomic validation. Successful deletion of each miRNA locus was confirmed by Sanger sequencing of PCR amplicons spanning the target region, and isoform-specific knockout was further verified by quantitative RT-PCR analysis.

### 4.3. RNA Extraction and Quantitative RT-PCR

Total RNA was extracted from SW48 parental, miR-196A-KO, and miR-196B-KO cells using TRIzol reagent (Invitrogen, Thermo Fisher Scientific, Waltham, MA, USA) according to the manufacturer’s protocol. RNA concentration and purity were assessed using a NanoDrop spectrophotometer (Thermo Fisher Scientific). For miRNA quantification, reverse transcription was performed using the TaqMan MicroRNA Reverse Transcription Kit (Applied Biosystems, Thermo Fisher Scientific, Waltham, MA, USA) with miRNA-specific stem-loop primers. Quantitative PCR was carried out on a StepOnePlus Real-Time PCR System (Applied Biosystems) using TaqMan MicroRNA assays. The expression levels of hsa-miR-196a-5p and hsa-miR-196b-5p were analyzed. U6 small nuclear RNA (U6 snRNA) was used as an endogenous normalization control. Relative expression levels were calculated using the 2^−ΔΔCt^ method. All reactions were performed in triplicate, and the results are presented as mean ± standard deviation (SD). To validate RNA-seq findings, quantitative RT-PCR was additionally performed for selected mRNA targets including NT5E, PRRX1, KITLG, CLDN4, and FLG. cDNA synthesis was performed using the High-Capacity cDNA Reverse Transcription Kit (Applied Biosystems, Thermo Fisher Scientific) according to the manufacturer’s instructions. Gene expression levels were normalized to *GAPDH* as an internal control. The primer sequences used were as follows: *NT5E* forward 5′-AGTCCACTGGAGAGTTCCTGCA-3′ and reverse 5′-TGAGAGGGTCATAACTGGGCAC-3′; *PRRX1* forward 5′-TGCTGCTCTACCTGCTCAAC-3′ and reverse 5′-GCTGTTGGTGATGTCCTTGG-3′; *KITLG* forward 5′-CTGCTCCTATGCTGCTCTTG-3′ and reverse 5′-TGTAGGCTTCCTTCTGCTGC-3′; *CLDN4* forward 5′-AGTGCAAGGTGTACGACTCGCT-3′ and reverse 5′-CGCTTTCATCCTCCAGGCAGTT-3′; *FLG* forward 5′-ACCTGGAGGTGGAGATGTTG-3′ and reverse 5′-TGGTCTTGGTGATGGTGTTG-3′; GAPDH forward 5′-GAAGGTGAAGGTCGGAGTC-3′ and reverse 5′-GAAGATGGTGATGGGATTTC-3′.

### 4.4. RNA Sequencing

For transcriptomic profiling, total RNA was extracted from three independent biological replicates of SW48, miR-196A-KO, and miR-196B-KO cells using TRIzol reagent (Invitrogen, Thermo Fisher Scientific, Waltham, MA, USA) as described above. RNA integrity was assessed using an Agilent 2100 Bioanalyzer (Agilent Technologies, Santa Clara, CA, USA), and only samples with an RNA integrity number (RIN) ≥ 7.0 were used for library preparation. Strand-specific RNA-seq libraries were constructed using the TruSeq Stranded mRNA Library Prep Kit (Illumina, San Diego, CA, USA) according to the manufacturer’s instructions, including poly(A) selection to enrich for mRNA. Libraries were sequenced on an Illumina NovaSeq 6000 platform using 150-bp paired-end reads, generating approximately 30 million reads per sample.

### 4.5. RNA-seq Data Processing and Differential Gene Expression Analysis

Raw sequencing reads were assessed for quality using FastQC (v0.11.9, Babraham Bioinformatics, Cambridge, UK) and trimmed to remove adapter sequences and low-quality bases using Trimmomatic (v0.39, RWTH Aachen University, Aachen, Germany). The trimmed reads were aligned to the human reference genome (GRCh38/hg38) using HISAT2 (v2.2.1). Gene-level read counts were obtained using featureCounts (v2.0.1) based on the GENCODE v42 gene annotation. Differential gene expression analysis was performed using the DESeq2 package (v1.38.0) in R (v4.2.0). Genes with an adjusted *p*-value (Benjamini–Hochberg correction) < 0.05 and an absolute log_2_ fold change greater than 0.585 (equivalent to a 1.5-fold change) were considered differentially expressed. Volcano plots and heatmaps were generated using the ggplot2 and pheatmap packages in R, respectively. The overlap of differentially expressed genes between miR-196A-KO and miR-196B-KO cells was visualized using a Venn diagram.

### 4.6. Hierarchical Clustering and Correlation Analysis

Hierarchical clustering analysis was performed using variance-stabilizing transformed (VST) expression values obtained from the DESeq2 package in R. Prior to clustering, genes with very low expression levels across all samples were filtered out to reduce noise and improve clustering accuracy. The VST transformation was applied to normalize count data and stabilize variance across the dynamic range of gene expression values. Unsupervised hierarchical clustering was conducted using the hclust function in R with Euclidean distance as the distance metric and Ward’s linkage method (ward.D2) for cluster aggregation. The resulting dendrograms were used to evaluate global similarities in gene expression patterns among SW48 parental, miR-196A-KO, and miR-196B-KO samples. Pearson correlation coefficients between samples were calculated based on VST-normalized gene expression values. The correlation matrix was visualized as a heatmap using the corrplot package in R to assess the overall reproducibility and similarity between biological replicates. In addition, pairwise scatter plots were generated to further evaluate the concordance of gene expression profiles between samples, allowing visualization of global expression relationships and potential outliers.

### 4.7. Gene Ontology Enrichment Analysis

Gene Ontology (GO) enrichment analysis was performed using the clusterProfiler package (v4.6.0) in R to investigate the functional characteristics of differentially expressed genes (DEGs). Differentially expressed gene lists derived from miR-196A-KO and miR-196B-KO cells were analyzed independently to identify potential functional differences associated with each knockout condition. Gene identifiers were converted to Entrez Gene IDs using the org.Hs.eg.db annotation database prior to enrichment analysis. Over-representation analysis was conducted using the enrichGO function with the human genome as the background gene set. GO terms from the Cellular Component (CC) and Molecular Function (MF) categories were analyzed. Statistical significance was determined using a Benjamini–Hochberg false discovery rate (FDR) correction, and GO terms with an adjusted *p*-value < 0.05 were considered significantly enriched. The enrichment results were visualized as bubble plots, where the bubble size represents the number of enriched genes associated with each GO term and the color intensity represents the −log_10_(adjusted *p*-value). These visualizations allowed intuitive comparison of functional enrichment patterns between miR-196A-KO and miR-196B-KO datasets.

### 4.8. Statistical Analysis

All quantitative RT-PCR experiments were performed with at least three independent biological replicates. Data are expressed as mean ± standard deviation (SD). Statistical comparisons were performed using Student’s unpaired two-tailed *t*-test in GraphPad Prism (version 9.0; GraphPad Software, San Diego, CA, USA). A *p*-value < 0.05 was considered statistically significant.

## 5. Conclusions

This study demonstrates that miR-196A and miR-196B regulate both overlapping and distinct transcriptional programs in CRC cells, with shared effects on extracellular matrix organization and epithelial keratin gene expression, and isoform-specific regulation of genes involved in metabolic reprogramming, neuroendocrine-like gene expression, and signal transduction. These findings establish a transcriptome-wide framework for understanding the isoform-specific biology of miR-196 in CRC and identify candidate downstream effector genes that may mediate the oncogenic activities of these miRNAs. The gene sets identified here provide a foundation for future mechanistic studies and may ultimately inform the development of miR-196-targeted diagnostic or therapeutic strategies for colorectal cancer.

## Data Availability

The data presented in this study are available in the article.

## Supplementary Materials

The following supporting information can be downloaded at: https://www.mdpi.com/article/10.3390/ijms27093959/s1.

## Abbreviations

The following abbreviations are used in this manuscript:

CC	Cellular Component
DEG	Differentially Expressed Gene
GO	Gene Ontology
KO	Knockout dichroism
MF	Molecular Function
miRNA	MicroRNA
RNA-seq	RNA Sequencing
SW48	Human Colorectal Cancer Cell Line SW48
VST	Variance Stabilizing Transformation
