# Supplementary material for: Transcriptomic Profiling Reveals Isoform-Specific Regulatory Roles of miR-196A and miR-196B in Colorectal Cancer Cells
Source: Int J Mol Sci. 2026 Apr 29;27(9):3959. doi: 10.3390/ijms27093959 (PMC13163813; doi:10.3390/ijms27093959)
Supplement: Supplementary file 1 [file ijms-27-03959-s001.zip › ijms-4261070-supplementary.pdf]

**Supplementary Table S1.** Quantitative enrichment statistics (gene count, gene ratio, fold enrichment, and adjusted p-value) for representative GO terms and KEGG pathways in SW48KO196A relative to SW48-vector cells

| Category | Term | Gene Count | Gene Ratio | Fold Enrichment | adj. p-value (FDR) |
|---|---|---|---|---|---|
| BP | Intrinsic apoptotic signaling pathway in response to ER stress | 8 | 0.10 | ~6.5 | 1.8E-07 |
| BP | Response to unfolded protein | 10 | 0.13 | ~7.5 | 1.1E-07 |
| BP | Protein folding | 9 | 0.11 | ~5.8 | 3.0E-07 |
| BP | Amino acid biosynthetic process | 7 | 0.09 | ~4.8 | 6.0E-07 |
| CC | Extracellular exosome | 15 | 0.18 | ~3.2 | 4.0E-06 |
| CC | Cytoskeleton | 12 | 0.15 | ~3.0 | 7.0E-06 |
| CC | Microtubule | 10 | 0.13 | ~4.5 | 3.5E-06 |
| MF | Identical protein binding | 14 | 0.17 | ~2.8 | 1.1E-05 |
| MF | ATP-dependent protein folding chaperone | 7 | 0.09 | ~4.2 | 5.5E-06 |
| MF | Unfolded protein binding | 9 | 0.11 | ~5.5 | 2.2E-06 |
| KEGG | Protein processing in endoplasmic reticulum | 11 | 0.14 | ~6.8 | 1.0E-06 |
| KEGG | MAPK signaling pathway | 9 | 0.11 | ~4.5 | 4.5E-06 |
| KEGG | PI3K-Akt signaling pathway | 8 | 0.10 | ~3.9 | 7.0E-06 |
| KEGG | mTOR signaling pathway | 6 | 0.08 | ~3.5 | 1.2E-05 |

**Supplementary Table S2.** Quantitative enrichment statistics (gene count, gene ratio, fold enrichment, and adjusted p-value) for representative GO terms and KEGG pathways in SW48KO196B relative to SW48-vector cells

| Category | Term | Gene Count | Gene Ratio | Fold Enrichment | adj. p-value (FDR) |
|---|---|---|---|---|---|
| BP | Response to unfolded protein | 7 | 0.11 | ~5.2 | 3.2E-05 |
| BP | Amino acid biosynthetic process | 6 | 0.09 | ~4.8 | 6.1E-05 |
| BP | Response to lipopolysaccharide | 5 | 0.08 | ~4.0 | 1.1E-04 |
| CC | Extracellular matrix | 8 | 0.13 | ~4.5 | 2.2E-05 |
| CC | Basement membrane | 7 | 0.11 | ~4.0 | 3.5E-05 |
| CC | Plasma membrane | 9 | 0.14 | ~3.6 | 4.1E-05 |
| MF | Structural constituent of ribosome | 6 | 0.09 | ~3.5 | 6.5E-05 |
| MF | Structural molecule activity | 7 | 0.11 | ~3.2 | 7.0E-05 |
| MF | Sodium channel activity | 4 | 0.06 | ~2.8 | 1.2E-04 |
| KEGG | Glycine, serine and threonine metabolism | 6 | 0.09 | ~4.2 | 5.0E-05 |
| KEGG | PI3K-Akt signaling pathway | 7 | 0.11 | ~3.8 | 6.5E-05 |
| KEGG | p53 signaling pathway | 5 | 0.08 | ~3.5 | 9.0E-05 |
| KEGG | Estrogen signaling pathway | 4 | 0.06 | ~3.0 | 1.3E-04 |

**Supplementary Table S3.** Top differentially expressed genes in miR-196 knockout SW48 cells

| Regulation | SW48KO196A/SW48-vector Gene | p-value | SW48KO196B/SW48-vector Gene | p-value |
|---|---|---|---|---|
| **Down** | *RIMS2* | 0.428 | *PCCA* | 0.136 |
| | *ADGRL2* | 0.436 | *LAMA2* | 0.392 |
| | *LAMA2* | 0.443 | *AKAP12* | 0.085 |
| | *MEF2C* | 0.406 | *SYTL2* | 0.000 |
| | *LINC01468* | 0.079 | *IGSF10* | 0.073 |
| | *INSIG1* | 0.032 | *ASMTL-AS1* | 0.017 |

| | | | | |
|---|---|---|---|---|
| | *UCA1* | 0.420 | *CEP152* | 0.015 |
| | *STARD4* | 0.238 | *STARD4* | 0.267 |
| | *SYTL2* | 0.012 | *CCDC186* | 0.139 |
| | *ASB4* | 0.438 | *PADI2* | 0.083 |
| **Up** | *KRT14* | 0.010 | *KRT16* | 0.005 |
| | *KLK11* | 0.001 | *KRT14* | 0.010 |
| | *KRT16* | 0.005 | *DDIT4* | 0.003 |
| | *FOXQ1* | 0.008 | *KRT19* | 0.018 |
| | *DDIT4* | 0.003 | *FAM129A* | 0.004 |
| | *FAM129A* | 0.004 | *SLC6A9* | 0.000 |
| | *CHAC1* | 0.000 | *FOXQ1* | 0.008 |
| | *LCN2* | 0.077 | *HKDC1* | 0.015 |
| | *SLIT1* | 0.000 | *NDRG1* | 0.032 |
| | *ADM2* | 0.006 | *CHAC1* | 0.000 |
